# Health System Challenges in Organizing Quality Diabetes Care for Urban Poor in South India

**DOI:** 10.1371/journal.pone.0106522

**Published:** 2014-09-04

**Authors:** Upendra Bhojani, Narayanan Devedasan, Arima Mishra, Stefaan De Henauw, Patrick Kolsteren, Bart Criel

**Affiliations:** 1 Institute of Public Health, Bangalore, Karnataka, India; 2 Department of Public Health, Institute of Tropical Medicine, Antwerp, Belgium; 3 Department of Public Health, Ghent University, Ghent, Belgium; 4 Health, Nutrition and Development Initiative, Azim Premji University, Bangalore, India; Rajarata Univeresity of Sri Lanka, Sri Lanka

## Abstract

**Background:**

Weak health systems in low- and middle-income countries are recognized as the major constraint in responding to the rising burden of chronic conditions. Despite recognition by global actors for the need for research on health systems, little attention has been given to the role played by local health systems. We aim to analyze a mixed local health system to identify the main challenges in delivering quality care for diabetes mellitus type 2.

**Methods:**

We used the health system dynamics framework to analyze a health system in KG Halli, a poor urban neighborhood in South India. We conducted semi-structured interviews with healthcare providers located in and around the neighborhood who provide care to diabetes patients: three specialist and 13 non-specialist doctors, two pharmacists, and one laboratory technician. Observations at the health facilities were recorded in a field diary. Data were analyzed through thematic analysis.

**Result:**

There is a lack of functional referral systems and a considerable overlap in provision of outpatient care for diabetes across the different levels of healthcare services in KG Halli. Inadequate use of patients’ medical records and lack of standard treatment protocols affect clinical decision-making. The poor regulation of the private sector, poor systemic coordination across healthcare providers and healthcare delivery platforms, widespread practice of bribery and absence of formal grievance redress platforms affect effective leadership and governance. There appears to be a trust deficit among patients and healthcare providers. The private sector, with a majority of healthcare providers lacking adequate training, operates to maximize profit, and healthcare for the poor is at best seen as charity.

**Conclusions:**

Systemic impediments in local health systems hinder the delivery of quality diabetes care to the urban poor. There is an urgent need to address these weaknesses in order to improve care for diabetes and other chronic conditions.

## Introduction

In India, chronic conditions are a leading cause of death and disabilities and estimated to account for 67% of all the deaths in the year 2020 [1]. The national prevalence of diabetes among 20–79 years old is 8.56%. With over 65.1 million people suffering from diabetes in 2013, India has the second largest number of people living with diabetes in the world, after China. Diabetes accounted for over one million adult deaths in 2013 [2]. Recent studies show that the major chronic conditions, including diabetes, are no longer the conditions affecting only the wealthy population but are increasingly affecting the urban poor and slum dwellers [3]–[8].

Weak health systems have been identified as major bottlenecks in effectively responding to the rising burden of chronic conditions in low- and middle-income countries (LMIC), including India [1], [9]–[11]. Despite recognition by global actors for the need for research on health systems [12], [13], little attention has been given to the role of local health systems in the delivery of care for chronic conditions. The local health system – defined as all organizations, people and actions that primarily intend to promote, restore or maintain health at the level of cities or rural areas – is key to health system performance. At this level, policies are adopted and implemented, responsive health services are provided and programs are applied. Recently, the integration of chronic disease prevention and management programs into district level health systems in India has been proposed [14], [15].

The study, presented in this paper, is part of a larger research project to understand how local health systems can be strengthened in order to deliver better quality chronic condition care to the urban poor. A poor urban neighborhood in South India constituted the research site. The research involved the following: (i) a household survey that revealed a high prevalence of diabetes and high out-of-pocket healthcare expenditure [8], [16]; (ii) interviews with diabetes patients that revealed specific constraints faced in managing diabetes [17]; and (iii) interviews with healthcare providers to better understand existing health system challenges in delivering diabetes care. This paper concerns the third aspect of this larger research framework.

## Methods

**Figure 1:**
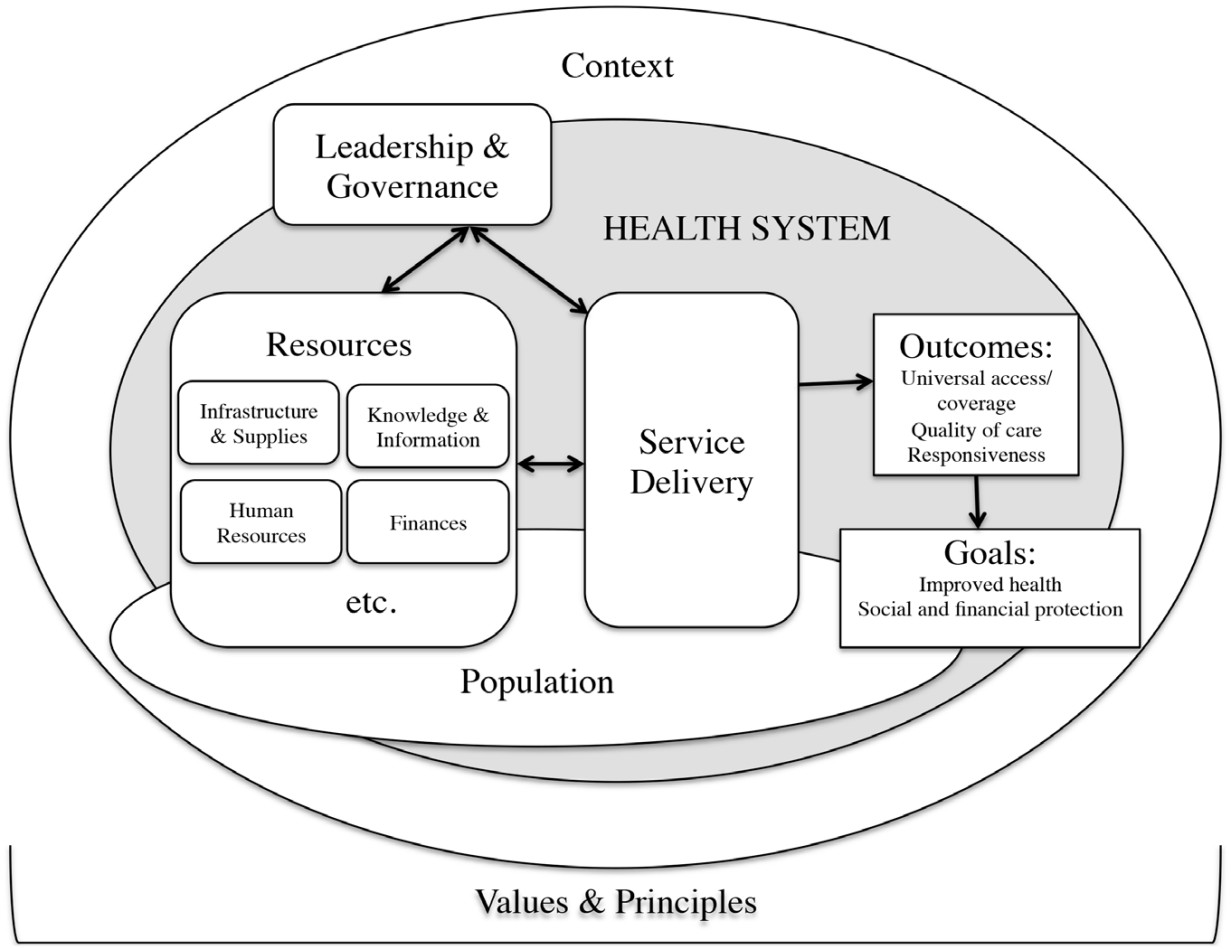
Health systems dynamics framework. The health systems dynamics framework developed by Van Olmen et al [18] at the Department of Public Health, Institute of Tropical Medicine, Antwerp, Belgium.

We conducted a cross-sectional study with healthcare providers providing care to patients with diabetes mellitus type 2 (referred to as diabetes in the rest of the paper). We used semi-structured interviews to understand the organization of diabetes care in the local health system and the problems in diabetes management, as well as to identify feasible health service interventions from the viewpoint of the healthcare providers. The enquiry was shaped by the health system dynamics framework developed by Van Olmen et al [18], [19] to analyze (local) health systems. This analytical framework (Figure 1) that includes ten interactive elements establishes the building blocks of health systems as specified by the World Health Organization (WHO) [20]. The framework also emphasizes that health systems should be geared towards outcomes and goals that are based on explicit choices of values and principles. The organization and delivery of healthcare services are considered the central processes and the immediate outputs of the health system. This framework has been helpful in analyzing local health systems in different contexts [19]. File S1 provides the detailed interview guides for doctors, pharmacists, and laboratory technicians in English.

The participants included specialist and non-specialist doctors, pharmacists and a laboratory technician working in and around Kadugondanahalli (KG Halli), a poor urban neighborhood in metropolitan city of Bangalore, the capital of the south Indian state of Karnataka. KG Halli is one of the 198 administrative units of Bangalore city with a slum area. KG Halli has a population of over 44,500 people within an area of less than a square kilometer.

KG Halli has a mixed health system with coexisting government and private healthcare sectors. Health facilities in KG Halli include two government health centers, 28 private clinics and four private hospitals. Clinics are primary care facilities managed by a single doctor who is occasionally assisted by support staff. Clinics operate on an outpatient basis. Hospitals, in addition to primary care, also provide specialist care. They provide facilities for surgery and inpatient care but greatly vary in size and services. In our study, we included all the health centers, clinics and hospitals in KG Halli that claimed to be offering care to diabetes patients and interviewed the doctors that used to treat diabetes patients at these facilities.

Our earlier study found that 85.2% of diabetes patients in KG Halli sought care from the private sector, often including health facilities located outside of the KG Halli area [8]. We therefore decided to include health facilities that were located within a two-kilometer radius (easy-to-travel distance) from KG Halli and were used by more than 50 diabetes patients from KG Halli as per our earlier study. Additionally, there are many private laboratories and pharmacies in KG Halli. We purposely selected one of each type from the frequently used private pharmacies and private laboratories and interviewed a pharmacist and a laboratory technician. We also interviewed a pharmacist in the government health center. Table 1 provides details about the health facilities included in the study.

**Table 1:** Profile of respondents.

Respondentnumber	Sex of therespondent	Role of the respondentat his/her healthfacility	Formal trainingof the respondent	Type of therespondent’s facility(based on ownership)	Type of the respondent’s facility(based on deliveryplatform)
R1	Woman	Non-specialist doctor	Graduation in ayurveda & bridging course in allopathy	Private	Clinic providing primarycare on outpatient basis
R2	Man	Non-specialist doctor	Graduation in unani	Private	Clinic providing primarycare on outpatient basis
R3	Woman	Non-specialist doctor	Graduation in unani	Private	Clinic providing primarycare on outpatient basis
R4	Woman	Non-specialist doctor	Graduation in unani	Private	Clinic providing primarycare on outpatient basis
R5	Man	Non-specialist doctor	Graduation in ayurveda	Private	Clinic providing primarycare on outpatient basis
R6	Man	Non-specialist doctor	Graduation in ayurveda	Private	Clinic providing primarycare on outpatient basis
R7	Man	Non-specialist doctor	Graduation in allopathy	Private	Clinic providing primarycare on outpatient basis
R8	Man	Non-specialist doctor	Graduation in allopathy	Private	Clinic providing primarycare on outpatient basis
R9	Man	Non-specialist doctor	Graduation in unani	Private	Clinic providing primarycare on outpatient basis
R10	Man	Non-specialist doctor	Graduation in allopathy	Government	Clinic providing primarycare on outpatient basis
R11	Woman	Specialist doctor	Post-graduation in allopathy	Government	Same facility as that of R10
R12	Woman	Specialist doctor	Post-graduation in allopathy	Private	Clinic providing specialistcare on outpatient basis
R13	Man	Non-specialist doctor	Graduation in allopathy	Private	Hospital (six beds)providing primary care and limited referral care
R14	Man	Non-specialist doctor	Graduation in unani	Private	Hospital (15 beds) providingprimary care and limited referral care
R15	Man	Non-specialist doctor	Graduation in ayurveda	Private	Hospital (30 beds) providingprimary care and limited referral care
R16	Man	Specialist doctor	Post-graduation in allopathy	Private	Super-specialty hospital(600 beds) attached to amedical school providingprimary and referral care
R17	Woman	Pharmacist	Graduation in pharmacy	Government	Same facility as that of R10
R18	Woman	Pharmacist	Completed schooleducation till 12^th^class	Private	Pharmacy
R19	Woman	Laboratory technician	Post-graduation in laboratory technology	Private	Diagnostic center

After acquiring respondents’ written consent, the first author, who has formal training and experience in qualitative research, conducted interviews in English or *Hindi* based on the respondents’ language preference. The data privacy and anonymity of respondents was assured. Considering the limited number of diabetes care providers in KG Halli, we explained to the respondents that they may be possibly identified by local actors and about the risk associated with it as part of the consent process. For respondents’ convenience, the interviews were conducted at their workplaces, usually in their consultation rooms. The interviews lasted approximately 40 to 60 minutes, occasionally interrupted by patient consultations. The interviews were tape-recorded and were transcribed verbatim by a professional transcriptionist. In addition to the interviews, the first author maintained a field diary recording the general observations made at the health facility while conducting the interviews. These observations included aspects such as physical environment of the health facility, writings and visuals displayed in the facility, and behavior of patients and staff at the facility.

We used thematic analysis. The first author coded transcripts in Nvivo software by creating respondents’ profiles and using elements of the health systems dynamics framework as tree nodes. Free nodes were created to accommodate data that did not fit the tree nodes. Based on the initial coding, the research team discussed the resulting overarching themes. They discussed the relationships across and between the themes and respondents’ attributes. The research team gathered expertise in relevant fields, including medicine, public health, health service research and medical anthropology. The major recurring themes were grouped into four categories representing the four out of the ten interactive elements of the health system dynamics framework (i.e., health service delivery, knowledge and information, leadership and governance, values and principles). We used these categories to present the study findings in the results section.

This study received approval from the Institutional Review Board at the Institute of Tropical Medicine, Antwerp (Belgium) and from the Technical Committee, as well as the Institutional Ethics Committee at the Institute of Public Health, Bangalore (India).

## Results

In total, we conducted 19 interviews with three specialist doctors, 13 non-specialist doctors, two pharmacists and one laboratory technician. These respondents were attached to a government health center, 11 private clinics, four private hospitals, a private pharmacy and a private diagnostic center. Table 1 provides the profiles of the respondents and their respective health facilities. A non-specialist doctor working at a private clinic refused to be interviewed whereas one non-specialist doctors and one specialist doctor who had agreed to participate and worked at a private clinic and a private hospital, respectively, did not have time for interviews despite repeated attempts by the researchers. Table 2 provides summary of dominant themes defining local health system challenges in delivering quality diabetes care.

**Table 2:** Dominant themes pertaining to local health system challenges in KG Halli.

Health systemelements	Dominant themes pertaining to challenges related to specific health system elements
**Health care delivery**	**Plurality in healthcare providers and care delivery platforms:** *KG Halli, with an area* *of less than a square kilometer, had several and diverse healthcare delivery platforms* *that catered to diabetes patients, most of which belonged to the private sector. Whereas* *doctors in the government health center were formally trained in allopathy, the doctors* *in the private sector were formally trained in different systems of medicine.*
	**Hospitals providing primary care:** *All the hospitals explicitly market for and provide* *basic primary care for diabetes in addition to providing the referral specialist care,* *creating a significant functional overlap with services provided by private clinics and* *the government health center.*
	**Private clinics delaying referrals:** *“One thing is that no one [doctor] wants to leave their* *patients. If a patient goes [referred to other facility], he may not come back. They* *[non-specialist doctors at clinics] have this fear.”* (R13, private hospital)
**Knowledge and** **information**	**Inadequate use of the patient medical records:** *Only six of the 15 health facilities in* *this study had a system that tracked medical records of diabetes patients.*
	**Periodically updating the knowledge of doctors & influence of pharmaceutical industry:** *Nearly half of all the doctors indicated that they periodically updated their clinical knowledge.* *The pharmaceutical companies had easy access to doctors for influencing their practice through* *personal periodic visits by company representatives, sponsoring of continuing medical education* *activities and provision of medical literature to doctors.*
	**Lack of standard treatment protocols:** *“No, there is nothing like that [standard treatment* *protocol]. It depends on how we analyze it [diabetes condition] and accordingly treat it.”*(R2, private clinic)
**Leadership and** **governance**	**Poor regulation of the private sector:** *“Many doctors in this area are not qualified to* *practice [allopathy]. But they have been doing it. … We have doctors who have a diploma* *in acupuncture and are practicing allopathy. Nothing is being done by the government.”*(R7, private clinic)
	**Poor systemic coordination:** *There was lack of coordination across different types of healthcare* *providers (government, private for-profit and not-for-profit) and across multiple health care delivery* *platforms (clinics, health centers, hospitals).*
	**Widespread bribery:** *“… It [kickbacks] happens in 90% of cases. It’s between pharmaceutical* *company and the doctor. This is rampant in this area.”* (R18, private pharmacy)
	**Lack of formal grievance redress platforms:** *Despite spending considerable amounts of money out of* *their pockets, the patients or community representatives had no formal functional platforms to engage* *with the formal healthcare services for expressing grievances, conveying opinions on issues or demanding accountability.*
**Values and** **principles**	**Maximization of profit:** *“It [healthcare] has become a business nowadays.”* (R6, private clinic)
	**Healthcare for poor as a charity:** *“We conduct the camps to test blood sugar for free to provide some* *services for those who can’t afford even sugar test.”* (R16, super-specialty private hospital)
	**Trust deficit among patients and providers:** *“Let the patient go to a physician. They will come back* *to you [non-specialist doctor] for small ailments. You should be happy because it is a circle. There* *should be no fear that if I send a patient to you, then tomorrow the patient will never come back to* *me. … I don’t think doctors have this kind of trust today”* (R1, private clinic)

### 1. Health Care Delivery

#### Plurality in healthcare providers and care delivery platforms

As enumerated in the methods section, KG Halli, with an area of less than a square kilometer, had several and diverse healthcare delivery platforms that catered to diabetes patients, most of which belonged to the private sector. All the clinics, some of the hospitals and a health center offered services for a specific duration in a day. Whereas doctors in the government health center were formally trained in allopathy, the doctors in the private sector were formally trained in different systems of medicine. In India, there are at least seven recognized systems of medicine apart from allopathy, grouped under the umbrella term AYUSH that refers to ayurveda, yoga, naturopathy, unani, siddha, homeopathy, and sowa-rigpa [21]. Of the 14 doctors interviewed in the private sector, four were trained in ayurveda, five in unani and six in allopathy (one non-specialist doctor had a dual training in ayurveda as well as allopathy). However, all of these doctors, irrespective of their training background, primarily practiced allopathy, which potentially compromised the competence of some of the doctors that were not formally trained in allopathy.

The doctors with training in ayurveda or unani learned to practice allopathy typically through reading literature and working early in their career in one or more of the private hospitals, where they observed practice by senior allopathic practitioners. In fact, all the allopathic hospitals but one had a duty doctor who graduated in ayurveda or unani.

*“After my studies [in ayurveda], I worked in XXX [a private allopathic hospital] as a duty doctor. I then worked in XXX [another private allopathic hospital] for six months in night shifts. … I watch the [allopathic] physicians and my senior doctors treat the patients. I will read the booklets. That is how I gained knowledge [about allopathy]”* (R15, private hospital).

When asked for their opinion, half of the doctors trained in allopathy believed that in the early and borderline cases of diabetes, ayurveda, yoga and naturopathy might play a supportive role, provided this care is done alongside provision of allopathic medicine and with strict blood glucose monitoring. Their support, at least in principle, for such ‘mix’ of medicines was due to the perceived harmful side effects of allopathic medication compared with the perceived safety of AYUSH practices and medications.

*“There are some very good medications in ayurveda that can be used for diabetes treatment for long time without harm. … However, one should control and monitor blood sugar well.”* (R10, government clinic).

*“For people with borderline diabetes, alternate medicines like, naturopathy, Ayurveda, yoga or homeopathy will do [work]. I welcome it. … If by using it, these [allopathic] medications could be reduced, it is good because in allopathy, there is ill in every pill but there is no pill for every ill.”* (R1, private clinic).

The doctors with training in ayurveda or unani also suggested that these systems have a very limited supportive role in early cases of diabetes and favored mixing this care with allopathy.

Despite the favorable attitude, none of the doctors exclusively trained in allopathy actually practiced mixed medicine. Two of the ayurveda and two of the unani-trained doctors occasionally, often because of patients’ demands, used ayurveda or unani medications along with allopathic medications in the treatment of early diabetes. Another non-specialist doctor with dual training in ayurveda and allopathy, who treated diabetes patients using allopathic medications, occasionally referred patients to doctors practicing AYUSH systems.

#### Hospitals providing primary care

In the private sector, unlike the government sector, there is no policy or plan to rationalize organization of care across different levels of health services. All of the hospitals explicitly market for and provide basic primary care for diabetes in addition to providing the referral specialist care, creating a significant functional overlap with services provided by private clinics and the government health center. In fact, more than 90% of all the patients being treated at private hospitals were walk-in patients that were not referred from other health facilities. This situation also occurred at the private super-specialty hospital and led to crowding in the outpatient department of the hospital. Based on different reasoning, a specialist doctor working at this hospital justified provision of primary care; he felt that if poor patients were refused care from his hospital (that provides subsidized care) on the basis that they needed to consult primary care providers, these poor patients would altogether fall out of the healthcare net. Furthermore, this doctor suggested that his fixed service-timings and fixed salaried remuneration meant that unlike specialists at other private hospitals working on fee-for-service basis, he did not have to be selective in terms of number of patients or kind of patients he sees.

#### Private clinics delaying the referrals

Hospital doctors believed that non-specialist doctors at private clinics do not refer patients in time and hold onto their patients until they can’t manage the patient anymore.

*“One thing is that no one [doctor] wants to leave their patients. If a patient goes [referred to other facility], he may not come back. They [non-specialist doctors at clinics] have this fear.”* (R13, private hospital).

Once patients were referred to hospitals, these patients were less likely to be referred back to clinics, as hospitals also provided primary care. This explains the apprehension of doctors at clinics about ‘losing’ patients by referring them to hospitals.

*“No, we don’t get [patients referred back from specialists/hospitals]. It [referral] is good but it depends on the specialist. … Once they [patients] go there [to specialists/hospitals], they will call them there only.”* (R3, private clinic).

### 2. Knowledge and Information

#### Inadequate use of the patient medical records

Only six of the 15 health facilities in this study had a system that tracked medical records of diabetes patients. Five of these six facilities (two private clinics, two private hospitals and a government health center) used patient-held, paper-based medical records for patients with chronic conditions such as diabetes or hypertension. This record was mainly in the form of a small booklet that could be conveniently carried by patients. In this booklet, doctors recorded information about investigations and medications during each encounter with patients. Patients were expected to bring the booklet to follow-up visits. Two of the facilities provided booklets to patients following the diagnosis, whereas patients were expected to purchase such booklets for themselves in the other three facilities. Only one of the five facilities had the corresponding facility-held, paper-based medical records for patients. Additionally, one specialist clinic used facility-held, electronic records with no corresponding patient-held records for patients with chronic conditions.

The patient-held medical records were advantageous, as they allowed for the continuity of information across health facilities/providers when patients sought care from other (than regular) facilities, including out of network or emergency facilities.

*“If a patient is staying far from this hospital or if a patient develops acute myocardial infarction, I don’t want him to waste his crucial time and come to me. He can go across to nearby health facility and show them all the treatment done till now [through his medical records].”* (R16, private super-specialty hospital).

The majority of the doctors, who did not use a medical record system, expressed its usefulness in improving clinical decision-making. They saw the record as useful because very few of their patients carried the loose medical prescription papers issued to them during earlier visits, making it difficult for doctors to make informed decisions. However, lack of time and the lack of human resources were reported as the common constraints for setting up and using a medical record system for patients.

*“They [patients] might bring the last prescription but not all [earlier prescriptions] … It [medical records system] will surely help but it is very difficult [for me] to get time to keep records.”* (R8, private clinic).

All the five hospitals that were studied used facility-held, paper-based medical records for hospitalized patients. During discharge from hospitals, the patients were provided with a discharge summary. None of the facilities, including those using medical record systems, had an active follow-up or reminder system for patients. Patients were lost to follow-up. Furthermore, there was no population-level routine surveillance system for assessing prevalence of diabetes or its risk factors.

#### Periodically updating the knowledge of doctors & influence of pharmaceutical industry

Nearly half of all the doctors indicated that they periodically updated their clinical knowledge. The common educational tools included the continuing medical education activities (seminars, lectures) organized by professional associations and reading medical literature. Five of the doctors were members of professional associations. They considered continuing medical education as the major activity of these associations that, one or more times, included diabetes as a topic.

The pharmaceutical companies had easy access to doctors for influencing their practice through personal periodic visits by company representatives, sponsoring of continuing medical education activities and provision of medical literature to doctors. Interestingly, two of the doctors reportedly used the internet as a source of learning the latest knowledge on diabetes management.

#### Lack of standard treatment protocols

Despite moderate participation in continuing medical education activities, none of the doctors except one specialist were aware of any standard treatment protocol for diabetes management. The management practice for diabetes varied across the doctors, beyond the adjustments needed to accommodate for the individual needs of patients.

*“No, there is nothing like that [standard treatment protocol]. It depends on how we analyze it [diabetes condition] and accordingly treat it.”* (R2, private clinic).

A few of the doctors, especially those not trained in allopathy, used a ‘trial and error’ approach for deciding on the use of allopathic medications promoted by pharmaceutical companies.

*“Once they [pharmaceutical companies] give [medication] samples, I try with patients. I will see the response, if it is good, okay, next time I will start with that. If patients don’t respond to it, then I send them to other [allopathic] doctors.”* (R4, private clinic).

Importantly, poverty in KG Halli has also shaped diabetes management practices of the doctors. Some of the doctors deviated from the knowledge-based clinical practices to adapt to the financial situation of patients even if the doctor knew the treatment would worsen the patient’s health status.

*“Most of the patients will come and ask for the [oral] tablets instead of insulin injections and we would give them tablets. … Insulin is costly and they have to take all these medications. If we are not doing it, somebody else [doctor] will do it [on patients’ request].”* (R14, private hospital).

### 3. Leadership and Governance

#### Poor regulation of the private sector

We used a limited interpretation of regulations by reducing them to the current toolbox of formal laws and policies. The laws formulated by governments to regulate healthcare, in the context of KG Halli, would require the following: (i) a healthcare provider to have a recognized qualification and a valid registration with the state council of her/his respective system of medicine; (ii) registration of private health facilities with the Karnataka Private Medical Establishment Act (2007) that prescribes the norms for healthcare infrastructure; (iii) a valid trade license for health facilities issued by the municipal government; and (iv) a No Objection Certificate from the Karnataka State Pollution Control Board that prescribes the norms for bio-waste management.

Of the 14 doctors working in the private sector, three knew about the regulation of bio-waste management, whereas six knew about the need for a trade license, as well as the registration of their facility, under some laws. The majority of them could not recall the name of the law or its major provisions. The doctors of only three of the private facilities (one clinic and two hospitals) were aware of all four regulations, and their facilities were in compliance with these regulations.

As mentioned earlier, the eight non-specialist private doctors, who had a degree in ayurveda or unani, primarily practiced allopathy without a degree or registration to do so. A pharmacist who ran a private pharmacy did not have the required degree. The majority of allopathy-trained doctors, who favored the mix of AYUSH with allopathy in treatment of early diabetes, were not supportive of doctors with AYUSH training that were primarily practicing allopathy.

*“Many doctors in this area are not qualified to practice [allopathy]. But they have been doing it. … We have doctors who have a diploma in acupuncture and are practicing allopathy. Nothing is being done by the government.”* (R7, private clinic).

Interestingly, most of the doctors in the private sector, including those who were not complying with the prevailing regulations, found these regulations meaningful in improving healthcare services. These doctors mentioned that the poor dissemination and enforcement of these regulations by government authorities was the major reason for non-compliance with regulations by private facilities. Another concern was the delay by regulatory authorities in processing applications and granting registrations/licenses.

*“They [regulations] are required and really good. But as far as the [enforcement] officers are concerned, I have not seen them coming down and checking it [compliance]. … We applied for the registration around two years ago, still we haven’t received any response.”* (R2, private clinic).

#### Poor systemic coordination

Beyond formal regulations, coordination across different healthcare providers (government, private for-profit and not-for-profit) and across multiple healthcare delivery platforms (clinics, health centers, hospitals) is an important regulatory mechanism to steer care providers towards a coherent vision and goal in the local health system.

However, basic information, such as the number of health facilities/doctors in KG Halli and the range of services they provide, is not collected by the government or any private player. Although each private facility is expected to provide information in a prescribed format to the appropriate government health authority in their area on a monthly basis, only one of the doctors in the private sector was aware of the process and had started doing so a few months prior to this study.

Government health workers or officers had never visited most of the private health facilities in the area. Some of the private doctors were not even aware of the location of a government health facility in the area.

*“Nobody [from government] comes here. Till now, in the last 20 years of my practice, I have not seen anybody from the government health service coming here.”* (R8, private clinic).

Within the private sector, only a few doctors who had been practicing for many years in the area knew the other doctors in the area and had a professional interaction with them. There was no coordination between two of the government facilities, which were located close to each other in KG Halli but managed by different government authorities. All of the super-specialty government referral hospitals (outside KG Halli) were managed by the medical education department of the state government with little or no coordination with the municipal authority or the health department of the state government that are supposed to manage primary care facilities in the city. There was no coordination between the government facility in KG Halli and the private facilities for planning the organization of diabetes care.

#### Widespread bribery

Bribery was common at both the individual healthcare provider and organizational level. The kickbacks from the pharmaceutical companies to doctors for writing particular brands of medication were commonplace.

*“… It [kickbacks] happens in 90% of cases. It’s between pharmaceutical company and the doctor. This is rampant in this area.”* (R18, private pharmacy).

In fact, one of the private health facilities in this study was owned by a pharmacist, who allowed three doctors to use that facility to practice without paying any rent or facility costs if these doctors directed their patients to his pharmacy housed in the same building. Interestingly, a private hospital had put a board in the patients’ waiting area that stated doctors at that hospital do not insist patients buy medications from any specific pharmacy. This message was to reassure patients who knew that health facilities often associate with specific pharmacies for kickbacks. Similarly, kickbacks from private diagnostic centers to the doctors in the area were common. In fact, some of the doctors used prescription papers that had the details of a specific private pharmacy or laboratory printed at the bottom of the papers.

*“We have to pay some 25 to 30 percent [of cost of prescribed investigations as a kickback] to doctors. We are giving percent to more than 20 to 25 doctors in this area.”* (R19, private laboratory).

Three of the private doctors, who were approached by government regulatory authorities, reported that it was common for doctors to bribe the lower-level government officers to get the necessary license/registration for their facilities or to avoid punitive actions.

*“The inspector had come to me. Mine is an eight feet by eight feet clinic [smaller than the minimum space needed to run a clinic by law]. I said, what to do sir? I am practicing here for the past 20 years. Where will I go now? Take 5000 rupees [∼ USD 83] sir. That drug inspector will not come for another year. Every year go on bribing them, go on practicing.”* (R1, private clinic).

#### Lack of formal grievance redress platforms

Because the highly utilized private health sector works on a fee-for-service basis, patients are the major funders of the health system. Despite spending considerable amounts of money out of their pockets, the patients or community representatives had no functional platforms to engage with the formal healthcare services for expressing grievances, conveying opinions on issues or demanding accountability. Such engagement happened rather informally to a very limited extent as part of the doctor-patient interaction during the medical consultation. In fact, one of the doctors in the private sector felt that patients prefer to use his hospital because they could personally hold the doctors or other staff accountable because they had paid for services.

### 4. Values and Principles

In mixed health systems, often characterized by a relative lack of stewardship, identifying common values that generally guide the health system is often difficult and not anticipated. We attempted to highlight the values and principles that were often mentioned by the healthcare providers during interviews that they believed to be important in shaping current medical practice, especially in the private sector.

#### Maximization of profit

Of the seven respondents who were willing to talk about the guiding factors of current medical practice in the private sector, all but one mentioned that healthcare has become a business in which the medical practice aims to maximize profits. Money, and not the patient, is at the center and guides the practice. Other respondents either refused to express their opinion or had no specific comment on this aspect. The respondents believed that in the past, doctors saw healthcare delivery more as a service to mankind that should yield a decent income for doctors. However, healthcare is increasingly becoming like any other business in which profit drives practice. This transition was seen as part of the larger societal transition in which money is becoming an important preoccupation in the lives of people in all sectors and not limited to healthcare.

*“It [healthcare] has become a business nowadays.”* (R6, private clinic).

An allopathic doctor referred to the very expensive medical (allopathic) education system, especially in the private sector, in which admissions to the medical schools are literally being purchased with huge sums of money, which then forces these medical graduates to ‘recover’ finances by charging more to the patients.

#### Healthcare for poor as a charity

When patients had difficulty affording healthcare, the common response from private providers was to refer them to a government facility. However, when the required treatment was available within their facilities, three of the private providers waived some of the treatment cost or gave free medications. Five of the doctors in the private sector, who expressed concern towards the poor economic conditions of patients, suggested the need to consider paying capacity of patients as a guiding factor in deciding the fees. The doctors believed in charging more money to patients who could pay and help those patients who can’t afford care by charging them less. Apart from waivers in treatment cost, some doctors organized free diagnostic health camps, occasionally with limited supplies of medication, as their way ‘to help’ poor patients.

*“We conduct the camps to test blood sugar for free to provide some services for those who can’t afford even sugar tests.”* (R16, super-specialty private hospital).

#### Trust deficit among patients and providers

Private doctors had strong negative opinions about government health services. Coupled with poor coordination between these sectors, this division led to low levels of trust. Implicit with the notion of referring poor patients to government hospitals and wealthier patients to private hospitals, the doctors believed strongly that government hospitals are poor facilities meant for poor patients. The overcrowding, long waiting times, scarcity of doctors, inadequate time and explanations provided to the patients, negative and even abusive attitudes of health workers, and lack of guidance to navigate chaotic set-ups in large hospitals shape the perceptions about government health facilities. Doctors in the private sector were referring poor patients to government hospitals, but they were not sure whether these patients would receive the needed treatment in the government hospitals.

*“If the patient is very poor, we refer them to XXX [government hospital] or some other government hospital. Otherwise, if the patient can afford, we will send them to a private hospital. … \A private hospital will give good services, they will not shout at any patients.”* (R14, private hospital).

However, a few autonomous super-specialty hospitals (for heart conditions, cancers) were perceived to provide similar care as the super-specialty private hospitals.

There was a lack of trust between the non-specialist and specialist doctors, which contributed to the poorly functioning referral system. As one of the non-specialist doctors working at a private clinic who struggled to make referral links work stated:

*“Let the patient go to a physician. They will come back to you [non-specialist doctor] for small ailments. You should be happy because it is a circle. There should be no fear that if I send a patient to you, then tomorrow the patient will never come back to me. … I don’t think doctors have this kind of trust today.”* (R1, private clinic).

The majority of doctors doubted and often blamed patients for failing to follow the prescribed treatment and lifestyle changes, and thought the patients were ignorant, unconscious, or illiterate.

*“There are many illiterate people in this area. They are not aware of things. I also see many educated people also who don’t follow (behavior change, medications). They are busy with other things and they are not conscious about it.”* (R9, private clinic).

## Discussion

### The analytical framework

Our study adds to the early experiences [22], [23] of applying the health system dynamics framework in LMIC. The framework was useful in shaping the research enquiry and the data analysis from a health system perspective. It helped to investigate the systemic impediments that affected the effective delivery of quality diabetes care, as well as the interconnectedness of various elements of the local health system, e.g. a specific financing strategy (fee-for-service) affecting doctors’ behaviors (more patients with short consultation time, no time for record keeping) that therefore affected healthcare (less attention to prevention and patient-centeredness in care, lack of medical history affecting clinical decisions) in a context guided by changing deontological and professional values (maximization of profit from medical practice) in many of the private health facilities.

However, designing research that enables use of the full scope of this framework is difficult. The broader scope of the health system (and the framework) that involves many actors/elements and their interrelationships poses challenges in sampling the respondents and designing the tools that help capture all the relevant information. In our study, respondents were limited to diabetes care providers, who mostly owned and managed their own health facilities. This reduced somewhat the complexity of our research, but was at the same time a limitation of our study, as it provides an analysis of the local health system from the viewpoint of only one set of actors. As explained in the introduction section, our earlier work investigated the perspectives of diabetes patients [17]. The use of indirect questioning in the interviews and recording of the observations at the health facilities helped us to better understand the issues that respondents would either hesitate to discuss or provide short answers that were difficult to be taken face value. This method particularly helped to investigate values and principles, kickbacks and patients’ participation in health services.

### Systemic impediments in diabetes care delivery

The major gaps in organizing diabetes care identified by our study were related to the four elements of the local health system: health service delivery, information and knowledge, leadership and governance, and values and principles guiding the system.

Some of the problems identified in our study have been ailing the urban health systems in India for many years. The government of India, in one of its national five year plans developed nearly three decades ago that included a discussion of non-communicable diseases for the first time, mentioned that “the organized referral services are almost non-existent” in urban areas [24]. The government health centers in urban India that were largely established to deliver family planning services in the context of the population control initiatives [25]–[31] lack provision of comprehensive primary care, including care for chronic conditions. This factor leads to (highly inefficient) overcrowding of tertiary hospitals. The multitude of government agencies providing healthcare at different levels of health services poses considerable coordination challenges. There are at least seven government agencies providing healthcare in Bangalore city, and there is very limited functional integration and no administrative integration across the agencies. The coordination between the government and the private sector is currently still a largely utopian task.

The regulation of the private health sector, which forms the dominant part of the healthcare delivery system in urban areas, is yet another challenge. Despite the acknowledgement of the need to regulate the private sector since the early independence period [30], except for a few regulatory initiatives, the federal government enacted legislation to regulate the private medical establishments in 2010 [32]. However, like our findings from the KG Halli demonstrate, the enforcement of and the compliance with the various private sector regulations remain dismal [33]. Although the organized bodies representing the private healthcare providers have often resisted formal regulation of the private health sector [34], [35], our study revealed that individual private healthcare providers (most did not comply with current regulations) perceived the existing regulations as meaningful for ensuring better healthcare services. Instead, they blamed the apathy of the regulatory agency for the poor compliance by the private sector.

However, there is more to the (local) health systems than formal regulations and health programs, many of which are formulated at higher (state or national) levels in India. As Gilson [36] states, “health systems are inherently relational and so many of the most critical challenges for health systems are relationship and behavior problems”. The actors within the local health system possess discretionary powers that, through their daily practice and action, shape healthcare reforms, including leadership and healthcare delivery in local health systems. Our study revealed that the limited coordination between the government and the private healthcare sector in KG Halli happened in a context in which government reached out to private doctors (e.g., trying to convince them to adhere to the tuberculosis treatment guidelines as specified by the national tuberculosis control program or providing interested private providers with vaccines and contraceptive devices to enhance delivery of preventive and family planning services). In addition to the legislative measures to regulate the private sector, which seem to be poorly enforced and complied with, proactive interactions between government and private healthcare providers could possibly enhance coordination and rationalization of care within local health system. The presence of government-initiated disease/condition programs and insurance schemes provide ‘entry points’ for engagements that could, with improving relationships over time, broaden the scope beyond the programs/schemes.

The present study, as well as our earlier work [17], revealed the poor relationships across healthcare providers, as well as between healthcare providers and patients in KG Halli, which contributed to a general lack of trust. We join Gilson [36] in arguing that the government needs to play a role beyond being the provider, funder or regulator of health services to manage the relationships and processes that influence the building of trust within local health systems. This goal could involve fostering interactions among and across government and private healthcare providers working in the area; developing a collective (health) vision for the community; and sharing of information and plans by health facilities that encourages complementary, if not joint, planning. Studies exploring the enhancement of leadership and management using similar modalities in a mixed urban local system in South Africa have shown encouraging results [37].

An important limitation of our study is that we analyzed the local health system of a relatively small poor urban neighborhood. The findings related to the health system challenges can therefore not be generalized to Bangalore city or to other areas in India. However, our findings imply the need for systems thinking in the planning of health programs for diabetes or other chronic conditions. Analytically, our study findings would help designing enquiries to understand systemic challenges in delivering care for chronic conditions in LMIC that are facing rising burden of chronic conditions and share some common challenges in their mixed health systems [9], [38]. The national program for diabetes, which is being piloted in selected districts across India, introduces some ‘new’ (preventive and curative) services within district health systems for diabetes patients, but there is still an overlap in care across different levels of health services [15]. The program aims to integrate diabetes care delivery into routine government health services at various levels, but it does not attempt to address many of the known systemic weaknesses in the existing government health system that are so critical to chronic condition care (e.g., poor information systems, lack of medications in government facilities or non-integration among and across government and private healthcare providers).

The government of India has recently launched the National Urban Health Mission, which is a population-based program to improve the health status of the urban population in general [39]. The programs focuses on the urban poor and disadvantaged and proposes a series of health system reforms addressing many of the gaps highlighted in our study, such as strengthening urban primary healthcare services including chronic condition care; enhancing referral links across different levels of healthcare services; and creating institutional platforms for community participation in health services.

Despite acknowledging the high utilization of large private health services by the urban poor, the mission largely focuses on the government sector. It does not include the private sector (the so-called ‘elephant in the room’) in discussing reforms in the government sector. The 89-page implementation framework dedicates a little over one page to mention the regulation of the health system [39]. The proposed regulations, including the development of quality standards for health services, the accreditation of health facilities and setting up of mechanisms for addressing user grievances, are all meant for the government facilities and for the limited number of private facilities that the mission might purchase. This indicates that the government does not have a strong will to regulate the private health sector. We strongly advocate that the government should take a broader and more inclusive view of the health system and augment the stewardship of the entire health system. We hope that the autonomy accorded to state and municipal governments in contextualizing the mission plan will consider the challenges as well as the potential of local health systems to enhance healthcare delivery, including diabetes care.

## Conclusions

There is a lack of functional referral systems and a considerable overlap in provision of primary diabetes care across the different levels of healthcare services. Inadequate use of patient medical records and lack of standard treatment protocols affect clinical decision-making. The poor regulation of the private sector, the lack of coordination among and across the government and private healthcare providers, the widespread bribery practices and the absence of any formal grievance redress platforms, reflect weak leadership and governance. There is a huge trust deficit between patients and healthcare providers. The private sector, in which the majority of healthcare providers lack the required training, is guided by profit maximization in which healthcare for poor people is, at best, seen as charity. These systemic impediments in local health systems hinder the delivery of quality diabetes care to the urban poor. Our findings indicate the urgent need to address these systemic weaknesses in local health systems in order to integrate and improve the care for diabetes and other chronic conditions.

## Supporting Information

File S1 **Interview guide for semi-structured interviews with healthcare providers.**
(PDF)
