# Supplementary material for: Health System Challenges in Organizing Quality Diabetes Care for Urban Poor in South India
Source: PLoS One. 2014 Sep 4;9(9):e106522. doi: 10.1371/journal.pone.0106522 (PMC4154689; doi:10.1371/journal.pone.0106522)
Supplement: File S1 — Interview guide for semi-structured interviews with healthcare providers. (PDF) [file pone.0106522.s001.pdf]

Guide for conducting semi-structured interview with medical practitioners in KG Halli

[The purpose of this interview is to map organizational characteristics of local health systems (governance, resources, coordination, and values/principles) in KG Halli and to understand possible health service interventions to improvement in quality of care from health providers' perspective along with factors that may facilitate or hinder implementation of such interventions]

(blue text – instructions, green text-guiding questions, red text-suggestive probes)

I. Introduction

Greet the doctor and thank him/her for giving appointment for interview.
Introduce yourself (interviewer) and Institute of Public Health, if interviewee is not already familiar with you.
Provide the interviewee with the leaflet on the study design and briefly explain about the study.
Explain about confidentiality and use of the study outcomes.
Introduce the consent form. Ask for consent to audio recording and note taking.

II. Interview

Start by asking some general questions that interviewee would be confortable to answer…

So, sir, tell me something about your practice…since how long are you practicing here?
(In case of long practice) How different this area was when you had started the practice?
Collect basic demographic data of participant (name, gender, age, religion, caste/tribe)
Do you have other clinics? Or visit other hospitals?
What are the usual reasons for which people come to your clinic?
How many people with diabetes you see in a month (or in a week)? Tell me about their general profile (age, economic background, disease stage etc.)

Depending on the issues under conversation, a shift can be made to any of the relevant inquiry theme below. Ensure that all the themes are covered in the interview. Any new theme/concept brought in by interviewee shall be accommodated and be probed if deemed relevant.

Continuity and coordination in healthcare

How regularly diabetic patients come for follow-up? (If there are concerns expressed regarding regularity of follow-ups) Why you think they do not come for regular follow-up?
What are other clinics/hospitals in this area that also provide diabetes care? How well you know those?
Do you receive patients through referrals? Or do you refer patients to others? Can you describe how this referral process work?
[Reasons for referrals, what factors determine choice of referral facility, counter referrals, sharing of information between facilities/providers involved in referral process]
(For private providers) Are you aware of government health facilities in this area? Do you interact with them? (Ask government providers similar question about private providers)
[Sharing information -through prescribed reporting format- among government and private providers]
Are you familiar with government authorities in this area (e.g. ward councilor, health inspector, link workers of health center etc.)?
What are your views regarding role of alternative medicines in diabetes management?(When interviewing alternative medicine practitioners, ask similar question about allopathic medicines)

Availability and use of information systems

Please describe the kind of data/information you collect regarding diabetics. How do you do this?
[For whom: outpatients/inpatients. What kind of data: individual/family profile, risk factors – tobacco use/exercise/diet, health status/outcomes, treatment. How: data remains with health provider/patients/both, individual card/family card/diary/registers/computer based system. If you are in doubt, request him/her to show relevant register/card]
How these data help you? (If no data is collected) Do you see any use of collecting data/information about your patients?
[For what use: monitor health status and treatment outcomes, shape ongoing practice, enhance person centered care, enable reminder services for patient follow-ups]
How has been your experience with this system? What kinds of challenges you face?

Support for self-management and prevention

Tell me sir, how much awareness you see among diabetics regarding lifestyle factors? What are specific lifestyle factors that you think are influencing diabetics in this area?
[Awareness about disease, risk factors, complications, self-management practices]
How do you deal with addressing these lifestyle factors?
[Patient education – Oral/education aid, how often, how long, on what aspects, by whom. Patient training – foot care, tobacco cessation, self-glucose monitoring, appropriate diet etc.]
What are the challenges you face while dealing with lifestyle issues? How you deal with these challenges?
Are you aware of any social services available in this area (like garden, help to patients for free medicines/food, counseling etc.) that can be of help to diabetics?
[Try to figure out how much of shared decision making -between provider and patient- happen in this process of dealing with lifestyle change]

Leadership and governance

Please describe all the regulatory requirements by government that you need to meet in order to provide health services in this area?
What are your views about these regulations? How meaningful are these regulations? Do providers take these regulations seriously? What are the challenges you face?
[If not mentioned, probe specifically about the Karnataka Private Medical Establishment Act, trade license from municipal health department, provider's registration with respective education council, certification from pollution control board etc.]
Can you tell me about rules/guidelines (if any) that staff at your clinic/hospital needs to follow in order to ensure accountable services? Who makes these rule/guidelines? How it is ensured that these rules are being followed?
Do you get approached by any government agencies in regard to health planning/programs? Is there any group/platforms where you meet with other providers to discuss about health? If yes, please give some details about that.

Human resources (availability, competence, motivation)

Please describe me, one by one, who all works in this clinic/hospital along with their qualifications/trainings, roles and responsibilities. Who reports to whom?
[Especially ask details on his/her (doctor) qualifications, year of graduation, experience etc.]
When a diabetes patient visits your clinic/hospital, what all procedure he/she goes through? Who all see him/her and do what?
Can you describe how you keep updating your knowledge/skills regarding medical practice or other aspects of your practice?
Did you or any of your team members undergo any specific training/courses on diabetes (or chronic disease) management? If yes, please provide details.

Are you aware of or following any guidelines for diabetes management? Please explain. What are the usual factors that you consider while prescribing a specific therapy to a diabetes patient?

Infrastructure and supply

List major infrastructure -building and equipment- (specifically for diabetes patients)

Please describe investigations facilities available at your clinic/hospital for diabetes patients. Where do you refer your patients for laboratory investigations? What are the factors that you consider while deciding referral laboratories? Are you aware of fees charged by referral laboratory for common tests that you prescribe for diabetes patients? Please give examples.
Do you dispense medicines at your clinic/hospital to diabetes patients? What are these medicines? (If no) How far patients have to go to but your prescribed medicines?
You prefer generic or branded medicines? Or both? What are you're your views in this regard (why branded? Generic? How you choose specific brands of medicines? What are the costs of brands you prescribe?
Do you think it is common for other doctors in the area to prescribe unnecessary or costly medicines? Please explain. Do majority of doctors follow any standard treatment guidelines?

Health care delivery

What are the timings of your clinic? How you came to fix these timings?
[What happens when patients need care beyond these timings?]
Do you provide outreach services or make home visits? Explain the reasons
Government health centers usually have responsibility for health of a defined population and so they usually think beyond patients seeking care in their clinics/hospitals. What are your views on such population focused approach? How would you describe private providers' approach in this regard?
Do you think that in KG Halli people from certain communities or religion go to certain health providers? What could be the reasons for this?
What are your views regarding fees charged to diabetics by health providers in this area? Are patients able to easily afford the fees charged?
[Ask for usual fees charged by interviewee's organization. What happens when patients express inability or difficulties in paying prescribed fees? Insist for specific examples. Do people prefer certain health providers depending on how much people earn (can pay)?]
What in your opinion are the main factors in terms of values/principles that guide private practice? (Or) government practice?
What, in your views, are the problems faced by diabetics in this area in regard to their healthcare?
What you think are possible changes that can be done by health providers in their practice that can improve healthcare for diabetics?
[Try to elicit specific examples of practice improvement. For each example, probe further to understand factors that can facilitate or hinder implementation of change in practice]

III. Closing

Switch off the recorder. Thank interviewee for time and inputs. Ask the interviewee if he/she wants to share anything. Assure the sharing of study results with interviewee. Ask permission to get back to interviewee for any clarifications/further information.

Guide for conducting semi-structured interview with pharmacist or laboratory technician (staff) in KG Halli

[The purpose of this interview is to map organizational characteristics of local health systems (governance, resources, coordination, values/principles) in KG Halli and to understand possible health service interventions to improvement in quality of care from health providers' perspective along with factors that may facilitate or hinder implementation of such interventions]

(blue text – instructions, green text-guiding questions, red text-suggestive probes)

IV. Introduction

Greet the pharmacist or laboratory technician and thank him/her for giving appointment for interview.
Introduce yourself (interviewer) and Institute of Public Health, if interviewee is not already familiar with you.
Provide the interviewee with the leaflet on the study design and briefly explain about the study.
Explain about confidentiality and use of the study outcomes.
Introduce the consent form. Ask for consent to audio recording and note taking.

V. Interview

Start by asking some general questions that interviewee would be confortable to answer…

So, tell me something about your work…since how long are you working here? When was this pharmacy/laboratory established?
(In case of long work history in the area) How different this area was when you had started working here?
Collect basic demographic data of participant (name, gender, age, religion, caste/tribe)
Do you own other pharmacies/laboratories?
What are the usual investigations for which people come to your clinic? How many people come to you to buy diabetes medicines or (blood sugar/urine sugar) investigations in a day (or in a week)?
Tell me about their general profile (age, economic background, disease stage etc.)

Depending on the issues under conversation, a shift can be made to any of the relevant inquiry theme below. Ensure that all the themes are covered in the interview. Any new theme/concept brought in by interviewee shall be accommodated and be probed if deemed relevant.

Infrastructure and supply

List major infrastructure -building and equipment- (specifically in reference to diabetes care)

Please describe various medicines or investigations (interviewer can use standard treatment guidelines for DM-2 to be able to enumerate or probe) available at your facility for diabetes patients.
How you get medicines or laboratory materials?
(In case of pharmacy) How you decide what all brands of medicines to keep? Do you keep generic medicines? Explain reasons.
[Try to understand how pharmaceutical representatives (from pharmaceutical companies), doctors' preferences, pharmacist's interests, paying capacity of people in the area or other factors affect this?]
Do you always have medicines demanded by patients available (or in stock)? Explain instances when patients demand medicines (or brand) that you do not keep? How you deal with this?
[Provision of alternative brands, frequency of stock outs, linking patients to another pharmacy etc.]

Service delivery

What are the timings of your pharmacy or laboratory? How you came to fix these timings?
[Compatibility with timings of nearby clinics/hospitals, what happens when patients need these services beyond these timings?]
Do you make home visits? Explain the reasons. (If yes,) describe how such visits are sought/coordinated?
How people come to your pharmacy or laboratory? Do medical practitioners refer them? Do they come on their own?
(In case of self-referrals) Please describe what kind of people come directly to you to purchase medicines or to undergo blood/urine sugar investigations? Why would you think they come directly to you without consulting their doctor? Is this common in this area? How you deal with such instances?
(In case of referrals from doctors) Do they bring any correspondence (prescription, investigation form etc.) from medical practitioner? Do you get patients referred by all the doctors in the area? Who frequently refers patients to your pharmacy or laboratory? Do doctors in this area suggest specific pharmacies or laboratories to their patients or do they leave it up to their patients to choose? What in your opinion affects patients' choice of specific pharmacy or laboratory?
[Choice of pharmacy or laboratory: doctors' referral, patients' own choice, distance of laboratory from clinic/hospital, others. Following the flow of the discussion, try to bring in the issue of kickbacks/incentives from pharmacies or laboratories to medical practitioners]
What are your views regarding fees charged by laboratories to do blood sugar (or urine sugar) investigations to diabetics in this area? OR what are your views regarding costs of medicines to be bought by diabetics? Are patients able to easily afford the fees charged?
(In case of pharmacy) Do patients buy all the medicines (for all the days) prescribed by doctors? (If not) How they decide what to buy and what not to buy? What kind of questions they ask you?
[Ask for usual fees charged by interviewee's organization. What happens when patients express inability or difficulties in paying prescribed fees? Insist for specific examples. Do people prefer certain health providers depending on how much people earn (can pay)?]
Do you get feedback/complains from patients using your services? Please explain what are the usual issues raised by patients? How you deal with that? Do you get feedback/complaints from doctors who refer patients to your pharmacy or laboratory? Please give examples of issues raised by doctors in past.
[Mechanisms to receive feedback/grievance of patients, to address these grievances. Dynamics of relationship between doctors, pharmacist/laboratory technician/patients]
What in your opinion are the main factors (or say values/principles) that underline how pharmacies or laboratories are functioning today? What your thoughts? Are you happy with how this works OR would you like to see any changes?
What, in your views, are the problems faced by diabetics in this area in regard to their healthcare?
What you think are possible changes that can be done by health providers, pharmacies, and laboratories in their practice that can improve healthcare for diabetics?
[Try to elicit specific examples of practice improvement. For each example, probe further to understand factors that can facilitate or hinder implementation of change in practice]

Availability and use of information systems

Please describe the kind of data/information you collect regarding patients and/or services you render. How do you do this?
[What kind of data: services rendered (laboratory investigations or medicines), personal information of patients, referring doctor. How: data remains with pharmacy/laboratory, shared with patient/doctor, registers/computer based system. If you are in doubt, request him/her to show relevant reports/registers]
How do you use these data? (If no data is collected) Do you see any use of collecting data/information about your patients?
[For what use: improving service management, retrieving old records, reminder to patients]
How has been your experience with this system? What kinds of challenges you face?

Human resources (availability, competence, motivation)
Please describe me, one by one, who all works in this pharmacy/laboratory along with their qualifications/trainings, roles and responsibilities. Who reports to whom?
[Especially ask details on his/her (pharmacist/laboratory technician) qualifications, year of graduation, experience etc.]
When a patient visits your pharmacy or laboratory, what all procedure he/she goes through? Who all see him/her and do what?
How do you keep yourself updated with new medicines coming in market OR other aspect of your work? Do you or your tem member undergo any training? If yes, please provide details.

Leadership and governance
Please describe all the regulatory requirements by government that you need to meet in order to provide health services in this area?
What are your views about these regulations? How meaningful are these regulations? Do providers take these regulations seriously? What are the challenges you face?
[If not mentioned, probe specifically about the Pharmacy Act, Clinical Drugs and Cosmetics Act, trade license from municipal health department, provider's registration with respective education council, certification from pollution control board etc.]
Is there an association or network of pharmacies or laboratories in Bangalore (area/state/national)? Are you part of such network/associations? What is the role played by such network/association?
Can you tell me about rules/guidelines (if any) that staff at your pharmacy/laboratory needs to follow in order to ensure accountable services? Who makes these rule/guidelines? How it is ensured that these rules are being followed?
Do medical practitioners in the area approach you? Do you approach them? What typically happens when a new pharmacy/laboratory is opened in the area? Is there any group/platforms where you meet with other providers to discuss about health? If yes, please give some details about that.

VI. Closing
Switch off the recorder. Thank interviewee for time and inputs. Ask the interviewee if he/she wants to share anything. Assure the sharing of study results with interviewee. Ask permission to get back to interviewee for any clarifications/further information.
